# Research progress of full electroluminescent white light-emitting diodes based on a single emissive layer

**DOI:** 10.1038/s41377-021-00640-4

**Published:** 2021-10-05

**Authors:** Hengyang Xiang, Run Wang, Jiawei Chen, Fushan Li, Haibo Zeng

**Affiliations:** 1 MIIT Key Laboratory of Advanced Display Materials and Devices, Institute of Optoelectronics & Nanomaterials, College of Materials Science and Engineering, Nanjing University of Science and Technology, Nanjing, 210094 China; 2 College of Physics and Information Engineering, Fuzhou University, Fuzhou, 350108 China; 3 Fujian Science & Technology Innovation Laboratory for Optoelectronic Information of China, Fuzhou, 350108 China

**Keywords:** Inorganic LEDs, Quantum dots, Photonic devices

## Abstract

Carbon neutrality, energy savings, and lighting costs and quality have always led to urgent demand for lighting technology innovation. White light-emitting diodes (WLEDs) based on a single emissive layer (SEL) fabricated by the solution method have been continuously researched in recent years; they are advantageous because they have a low cost and are ultrathin and flexible. Here, we reviewed the history and development of SEL–WLEDs over recent years to provide inspiration and promote their progress in lighting applications. We first introduced the emitters and analysed the advantages of these emitters in creating SEL–WLEDs and then reviewed some cases that involve the above emitters, which were formed via vacuum thermal evaporation or solution processes. Some notable developments that deserve attention are highlighted in this review due to their potential use in SEL–WLEDs, such as perovskite materials. Finally, we looked at future development trends of SEL–WLEDs and proposed potential research directions.

## Introduction

Artificial light sources have been closely related to human life and production activities since the first torch was lit 600,000 years ago. Light sources have also developed from the initial flame to electric light, such as incandescent lamps, fluorescent tubes, and white light-emitting diodes (WLEDs)^1–3^. Electric light sources originated within the past two hundred years and promoted the rapid development of human society because they were safer and more convenient. However, in recent years, lighting swallows 15% of global electricity and releases 5% of the world’s greenhouse gas emissions^4,5^, which is a huge obstacle to energy conservation and carbon neutrality. Developing efficient lighting technology and providing better light quality have become urgent global tasks. In 2014, the Nobel Prize in Physics was awarded to Nakamura, S. et al. for their outstanding contributions in gallium nitride (GaN)-based blue LEDs and WLEDs, which have a huge advantage in saving electricity and are becoming the main light source in lighting, displays, and other fields^6,7^. In recent years, WLEDs have made a great contribution to human society owing to their high brightness and efficiency, high colour-rendering index (CRI), and adjustable correlated colour temperature (CCT)^1,8^. What is more noticeable is that the development of WLEDs has not stopped even though WLEDs are already a low-cost, universal technology. Organic molecules, quantum dots (QDs), perovskite materials, and many other luminescent materials continue to appear and show advantages in electroluminescent devices, such as organic light-emitting diodes (OLEDs), quantum-dot light-emitting diodes (QLEDs) and perovskite light-emitting diodes (PeLEDs), bringing a variety of outstanding characteristics, such as ultrathin, flexible, and transparent properties. Therefore, they have been developed and have prospects in lighting, displays, wearable devices, and other applications, but they are limited by complicated manufacturing processes and high costs^9–11^. Considering these conditions, developing a low-cost lighting a simple structure will be one of the main directions in the future development of lighting technology. WLEDs based on a single emissive layer (SEL), as an ideal strategy, have been continuously researched following the development of OLEDs and QLEDs^12,13^. In a SEL–WLED, white light normally comes from the electroluminescence of a single emissive layer, which contains multicoloured emitting centres (e.g., red, green, blue, and orange) or the entire visible-light broad-spectrum luminescence. This means that the device structure is simpler than that of current commercial GaN-based WLEDs, and the device, therefore, has great advantages for many applications requiring an ultrathin structure and flexibility. In terms of device fabrication, when conventional WLEDs need to be stacked in two or three emitting layers, SEL–WLEDs can replace multiple emitting layers with a single layer, which can both simplify the manufacturing process and reduce the production costs, showing promising prospects in future lighting and other applications.

**Fig. 1:**
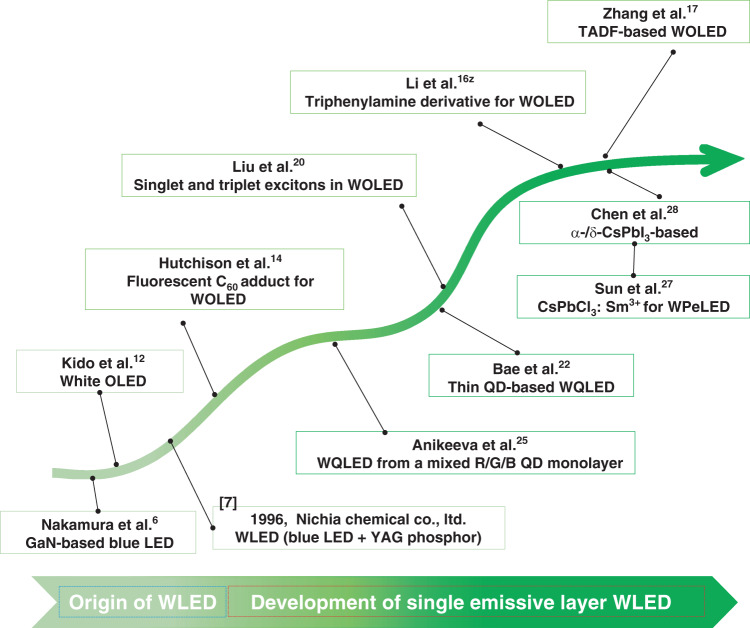
A brief LED and single emissive layer (SEL) WLED timeline

A brief LED/SEL–WLED timeline is listed in Fig. 1. At almost the same time as WLEDs with multilayer structures^12,13^, SEL–WLEDs began receiving extensive attention and are continuously developing. Some organic materials that emit blue/orange^14–17^ or red/green/blue (R/G/B)^18–20^ light are used to generate SEL–WLEDs. Some R/G/B QDs can also be mixed in an SEL^21–25^, showing potential in lighting applications, especially considering the advantage of their low-cost solution-process capability. Some very recent studies have confirmed that some perovskites can emit broadband white light^26–29^, and they have great potential for imitating sunlight, the ideal light source, and are expected to create future lighting technology.

**Fig. 2:**
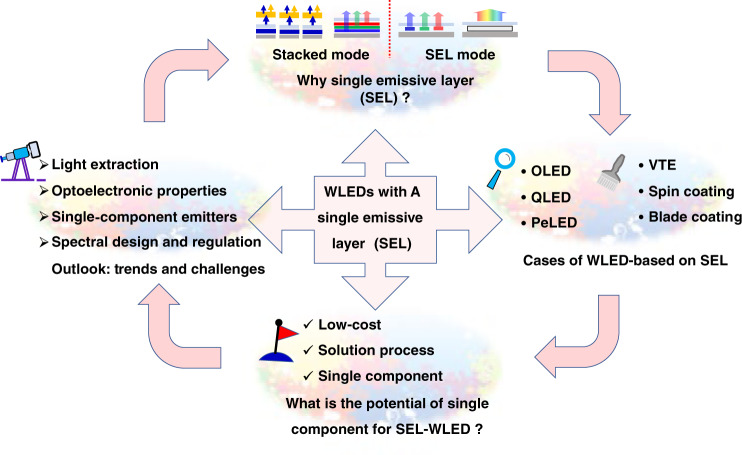
An overview of the discussion sections in this review

We reviewed the progress that has been made since these important developments occurred; we focused on the SEL–WLEDs in various emitters, device structures and their performance, hoping to spread these exciting results and promote research on white light sources. An overview of the discussion sessions in this review is shown in Fig. 2. We first introduced the emitters and the device structures employed in WLEDs because the electroluminescence characteristics, material features, and device structures are prerequisites for creating SEL–WLEDs and are also key points of white light technology. Then, we analysed the features of these emitters, such as organic molecules, QDs and perovskites, and the coelectroluminescence mechanisms of their multicoloured centres in SEL–WLEDs. Subsequently, we reviewed some cases of the above emitters that included vacuum thermal evaporation or solution processes. Some notable developments that deserve attention are emphasised owing to their potential for SEL–WLEDs, e.g., perovskite materials, which exhibit excellent photoelectric performance in both pure-colour emission (narrow peak) and broad-spectrum multicolour coemission. Finally, we discussed the outlook of the development process and trend of SEL–WLEDs and proposed future development directions, including the performance improvement, luminescence mechanisms, and the design of luminescent materials.

## Emitters and their electroluminescence mechanism in WLEDs

**Fig. 3:**
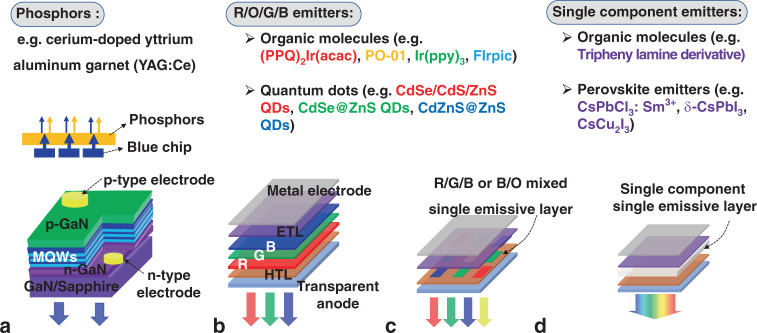
Emitters and device structures in WLEDs. Device structures of WLEDs based on **a** GaN–LED, **b, c** ROGB organic molecules/QDs, and **d** broad-spectrum emitting materials

The materials in the light-emitting layers play a vital role in the development of WLEDs (Fig. 1). Different luminescence characteristics lead to different light-emitting modes, as shown in Fig. 3. In WLEDs, WOLEDs, and WQLEDs, stacking and multicolour blending models are the main ways to achieve white light. In GaN-based WLEDs, blue LEDs combine with phosphors to form a WLED (blue–yellow stacking model, Fig. 3a), in which electroluminescence only comes from blue chips, and the generation of white light requires multicoloured phosphors with high-efficiency photoluminescence (PL)^30,31^. Furthermore, full electroluminescence can be achieved through WOLED/WQLED by multicolour mixing for white light generation because the colours of organic molecules and QDs are adjustable (Fig. 3b). The red/green/blue or blue/orange stacking model is commonly employed and contributes to high efficiency^32–34^. There is no doubt that OLEDs/QLEDs have advantages in energy consumption and colour rendering with additional flexibility potential. However, in regard to lighting, the cost of manufacturing and materials has always hindered the commercialisation of OLEDs/QLEDs^35–37^. High-efficiency white OLEDs rely on high-precision host–guest doping processes when creating the stacking layers; these processes require expensive organic materials and expensive vacuum thermal evaporation equipment. QLEDs have low-cost potential in luminescent materials, but the technology required to achieve high-efficiency white QLEDs cascades through the intermediate connection layer, meaning that the fabrication process is complex^34^. Therefore, material cost and device structure are considered to be the keys of lighting. As we mention in this review, an ideal solution is to implement WLEDs with simple structures, such as a single light-emitting layer, as a feasible and promising lighting technology. We review some WLEDs based on SELs with the feature of multicolour coelectroluminescent centres, for example, multicolour emitters for mixed SEL–WLEDs (Fig. 3c) and broadband-spectrum-emitting SEL–WPeLEDs (Fig. 3d).

**Fig. 4:**
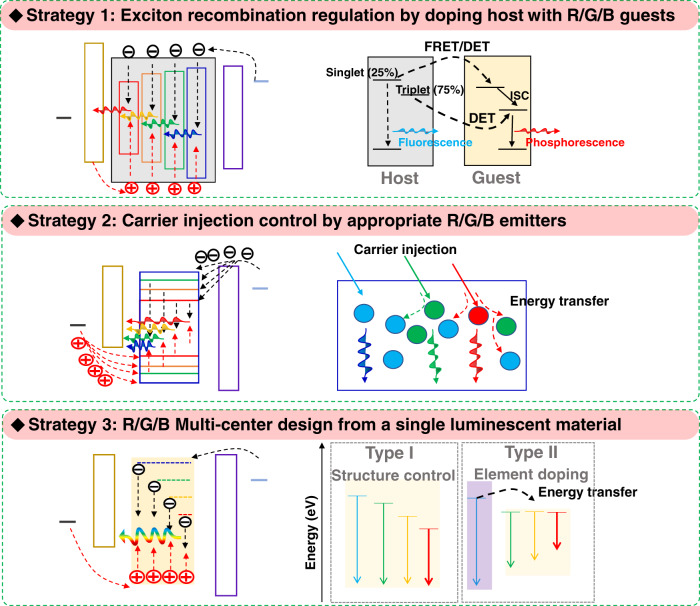
Strategies and their luminescence mechanisms for achieving multicolour coelectroluminescence in an SEL

In accordance with the emitters and device structures shown in Fig. 3c, d, we first analysed the strategies for achieving multicolour coelectroluminescence in a single light-emitting layer and summarised them in Fig. 4. Strategy 1 normally refers to the exciton recombination regulation between the cohost and R/G/B guests in SEL–WOLEDs^17,20^. A wide-bandgap material acts as the host material; in contrast, materials with narrow bandgaps that emit specific colours (e.g., R/G/B) can act as guests. In this mixed host/guest system, there are two important energy-transfer processes working for both effective luminescence and multicolour coelectroluminescence regulation: Fӧrster resonance energy transfer (FRET) and Dexter energy transfer (DET). As shown in the diagram on the right in Strategy 1 (Fig. 4), singlet excitons generated on the host (fluorescence) can transfer energy to the guest (phosphorescence) through the FRET or DET process, become triplet excitons by intersystem crossing (ISC), and then undergo radiative decay. In addition, the triplet excitons of the host can transfer energy to the guest via the DET process. The energy transfer process can be controlled by selecting matched materials as the host and guest materials, thereby improving the performance of the SEL–WLEDs, such as luminous efficiency and carrier balance in R/G/B guests. Simple mixing of multicolour emitters is another feasible strategy (Strategy 2 in Fig. 4), which has been demonstrated in some SEL–WQLEDs^22,25^. Due to the lack of ideal energy-transfer control in the mixed R/G/B QD layer, carrier injection preferentially reaches the low-energy red-colour centre, making it difficult to achieve efficient and balanced R/G/B multicentre coelectroluminescence. Concentration-adjustment methods are used to solve this problem. The concentration of red QDs is reduced, while the concentration of blue QDs is increased, so that the surplus carriers can be transferred from the red centre to the green/blue centre or from the green centre to the nearby blue centre, as shown in the right figure of Strategy 2 in Fig. 4. In a mixed R/G/B SEL, the presence of a single SEL-component material as SEL means that one material has multiple exciting centres, resulting in multicolour or broad-spectrum luminescence in the entire visible-light region. To avoid the above-mentioned problem of the balance regulation of carriers or excitons, it is necessary to design suitable materials in which multicolour centres can coexist. Some progress has been made in organic molecules and perovskite emitters through two design routes: structure control (Type I) and element doping (Type II), as shown in Strategy 3 in Fig. 4. For example, in some organic molecules^16,38^ such as triphenylamine derivatives, thermally activated rotation in the molecular structure leads to the enhancement of π–π stacking and electronic coupling, which thereby results in a redshift in some molecules. In some crystals, the lattice distortion-induced self-trapping state can also promote the coexistence of multicolour centres and has attracted much attention for some perovskite emitters in recent years, such as Cs_2_Ag_0.60_Na_0.40_InCl_6_, *δ*-CsPbI_3_, and CsCu_2_I_3_^26,28,29^. In addition to structural control, the introduction of new elements can often achieve new luminescence centres owing to their newly formed energy level. Through energy transfer from the intrinsic luminescence centre, doping elements can contribute to other luminescence centres, such as the red centre of Mn^2+^ and the yellow–green and red centres of Sm^3+^^27,39^. In this review, we relate these three strategies to typical cases and analyse their characteristics and potential in the lighting field, hoping to provide some inspiration for the future development of low-cost, highly efficient, ultrathin lighting technology.

## Cases of WLEDs with a single emissive layer

### WOLEDs with a single emissive layer

WOLEDs with a single emissive layer originated from the multicolour mixing strategy. As early as 1993, single-layer white light was developed by Kido J. et al., meaning that the single-layer white light strategy has always been of great value in the field of WLEDs^18^. In this SEL–WOLED, an SEL was fabricated by doping poly(N-vinylcarbazole) (PVK) with R/G/B fluorescent dyes. After this work, many WOLEDs with R/G/B or B/O mixed models emerged owing to the development of fluorescence and phosphorescence molecules with different colours (R/O/G/B). Due to the material properties of these organic molecules, the corresponding methods of forming SEL films have also been expanded to vacuum thermal evaporation^37–46^ and spin coating^47–55^, which are more convenient than multicolour stacking because they simplify the preparation process and even partially replace thermal vacuum evaporation.

**Fig. 5:**
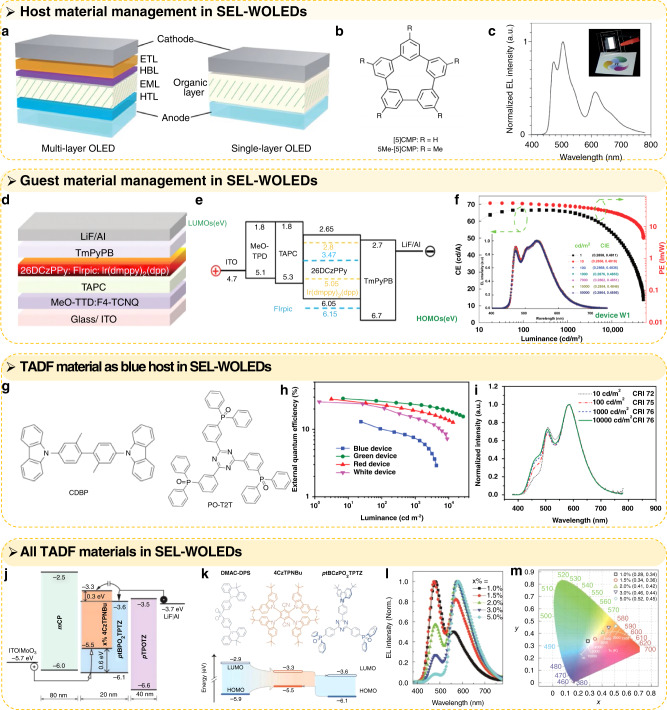
Management of carrier transport and exciton recombination in SEL–WOLEDs. **a** Architectures of electrophosphorescent OLEDs: a multilayer OLED with a four-layer architecture and a single-layer OLED with a simple architecture. **b** Chemical structures of the CMPs (cyclo-meta-phenylenes). **c** Electroluminescence spectrum at a current density of 0.1 mA cm^2^; inset: a picture of the device with an emitting area of 16.6 mm*6 mm. Reprinted with permission from ref. ^47^. Copyright 2016, The Royal Society of Chemistry. **d** The structure of SEL–WOLED (W1). **e** The proposed highest-occupied molecular orbital (HOMO) and lowest-unoccupied molecular orbital (LUMO) of the device. **f** Forward-viewing CE and PE as a function of luminance for device W1. Inset: normalised EL spectra along the whole range of luminance. Reprinted with permission from ref. ^46^. Copyright 2015, The Royal Society of Chemistry. **g** Molecular structures of CDBP and PO-T2T. **h** EQE-luminance characteristics of the devices. **i** EL spectra and CRI values of the hybrid WOLED at different luminances. Reprinted with permission from ref. ^20^. Copyright 2015, WILEY-VCH Verlag GmbH & Co. KGaA, Weinheim. **j** Device configuration of ITO/MoO_3_ (6 nm)/mCP (80 nm)/blue TADF emitter:4CzTPNBu (x%, 20 nm)/pTPOTPTZ (40 nm)/LiF (1 nm)/Al (100 nm). **k** Chemical structures and frontier molecular orbital (FMO) energy-level scheme of blue-emitting ptBCzPO2TPTZ and DMAC-DPS and yellow-emitting 4CzTPNBu. **l** EL spectra of ptBCzPO2TPTZ-based devices with different x%. **m** CIE coordinate dependence of the devices on x% and the corresponding positions on the CIE 1931 chromaticity diagram. The black-body locus and the colour-temperature lines were added for reference. Reprinted with permission from ref. ^41^. Copyright 2020, WILEY-VCH Verlag GmbH & Co. KGaA, Weinheim

To improve the light-emitting performance of SEL–WOLEDs, the management of carrier transport and exciton recombination in the SEL is essential, especially in phosphorescent organic molecule-based devices, which usually exhibit higher performance due to their efficient exciton utilisation with the highest internal quantum efficiency of 100%^56^. Therefore, a series of studies has focused on the development of host–guest-compatible materials and ways to simplify the device structure by reducing the number of materials in phosphorescent emitter-based SEL–WOLEDs. Many materials with bipolar characteristics have been simultaneously employed as hosts, electron-transport layers, and even hole-transport layers^17,40,47^. Xue J. Y. et al. designed and synthesised a multifunctional material for high-efficiency SEL–WOLEDs through simple one-pot macrocyclization (aromatic hydrocarbon component of toluene)^47^. Because it applied this multifunctional base material as the electron-transport layer, host material, and hole-transport layer, the device architecture was simplified to the extreme, as shown in Fig. 5a^47^. By doping this multifunctional base material (5Me-[5]CMP, Fig. 5b) with R/G/B phosphorescent emitters (R: Ir(piq)_3_, G: Ir(ppy)_3_, B: fac-Ir(mpim)_3_), the SEL–WOLED achieved an external quantum efficiency (EQE) of over 10%, and its white EL spectrum is shown in Fig. 5c. The results of many studies indicate that bipolar materials are conducive to more balanced charges and broader recombination regions, so that the devices have higher efficiency and better colour stability^17,40,47^. In addition to the host materials, the selection and optimisation of the guest materials is also very important in realising highly efficient and colour-stable SEL–WOLEDs. In their work, Liu B. et al. applied iridium(III)bis[(4,6-difluorophenyl)-pyridinato-N, C2] (FIrpic) and bis(2-phenyl-4,5-dimethylpyridinato) [2-(biphenyl-3-yl) pyridinato] iridium(III) [Ir(dmppy)_2_(dpp)] as blue and orange emitters; both the hole and electron mobilities were reduced, and the charges and exciton distributions were well controlled^46^. Therefore, the recombination ratio was more constant, and the device structure and energy level are shown in Fig. 5d, e. Thanks to the optimisation of the guest material and the design of the host material and device structure, this SEL–WOLED can achieve a high-power efficiency (PE) of 75.5 lm W^−1^ at 1000 cd m^−2^ and extremely stable colours (colour variation = (0.00, 0.00)), as shown in Fig. 5f.

In addition to conventional fluorescent and phosphorescent materials, some thermally activated delayed fluorescent (TADF) materials have also been introduced into SEL–WOLEDs^20,41^. To overcome the low performance of the SEL–WOLEDs based on traditional blue fluorescent hosts, a TADF blue exciplex (CDBP:PO-T2T) was introduced into the emitting layer, showing advantages in both efficient blue TADF emission and triplet hosts for green/red phosphors^20^. The TADF molecular structure is shown in Fig. 5g. The SEL–WOLED that using the TADF blue exciplex system can achieve a maximum PE of 84.1 lm W^−1^ and EQE of 25.5% at a low turn-on voltage (2.5 V), the EQE and white EL spectra are shown in Fig. 5h, i. In addition to partial replacement by TADF materials, a full-TADF SEL–WOLED with highly efficient and colour-stable features is demonstrated by doping a blue TADF matrix (ptBCzPO2TPTZ) with a yellow TADF emitter (4CzTPNBu). The energy level and carrier transport of the device, the chemical structures, and the frontier molecular orbital (FMO) energy-level scheme of blue-emitting ptBCzPO2TPTZ and yellow-emitting 4CzTPNBu are shown in Fig. 5j, k^41^. Through adjustment of the doping ratio (X% = 1.5%, 2.0–3.0%, and 5.0%), the SEL–WOLEDs can emit cool white light with correlated colour temperatures (CCTs) of 8332 K, pure white light with a CCT of 5152 K, and warm white light with CCTs of 3563 K and 2883 K. Electroluminescence spectra, CIE coordinates, and real photographs of these devices with the respective doping ratios are shown in Fig. 5l, m. TADF materials are regarded as low-cost, highly efficient third-generation organic light-emitting materials because they can surpass the internal quantum efficiency (25%) limit of regular fluorescent materials, while most phosphorescent materials contain some indispensable heavy metal atoms (e.g., Ir, Pt, Pd, and Os) and therefore face high cost and toxicity issues^57–59^.

**Fig. 6:**
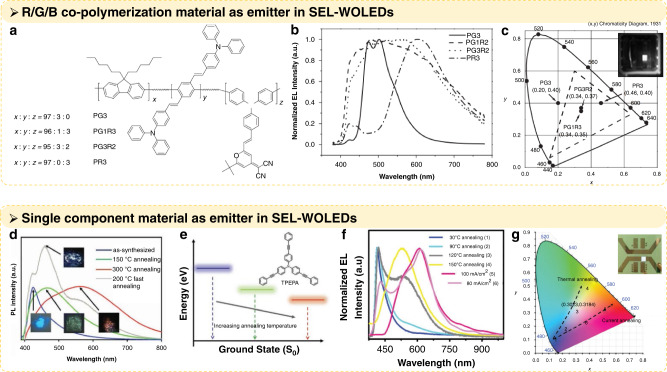
Applying single substance emitters for SEL–WOLEDs. **a** Molecular structures of fluorene-based copolymers. **b** EL spectra of the PG3, PG1R3, PG3R2, and PR3 devices with ITO/PEDOT:PSS/polymer/Ca/Al configurations under 9 V. **c** CIE coordinates (x, y) of the PG3, PG1R3, PG3R2, and PR3 devices under 9 V (high-definition, HDTV, dashed line). Reprinted with permission from ref. ^48^. Copyright 2005 WILEY-VCH Verlag GmbH & Co. KGaA, Weinheim. **d** PL spectrum of synthesised TPEPA annealed at 150 °C and 300 °C and fast-annealed at 200 °C. The insets are images of these four powders under a 380-nm UV–LED source. **e** Schematic energy-band diagram of the PL spectrum with increasing annealing temperature. The inset is the molecular structure of TPEPA. **f** Normalised EL spectra of these six OLED devices. **g** CIE colour coordinates of these six OLED devices. The dashed line indicates that the possible colour region formed by this single organic material and the CIE coordinates of white OLED devices is (0.3023, 0.3184). Inset photo refers to the WOLED based on 120 °C annealing. Reprinted with permission from ref. ^16^. Copyright 2019 WILEY-VCH Verlag GmbH & Co. KGaA, Weinheim

Compared with the multicolour emitter mixing strategy, a single substance emitting white light has a more obvious advantage in achieving SEL–WOLEDs because it would eliminate the need for high-precision doping and the complicated R/G/B mixing ratio of the above devices. Some studies have tried to load units that emit different colours of light on the same material, so that a single substance can emit white light^48,50,51,54,60,61^. Lee S. K. et al*.* designed fluorene-based copolymers by the polymerisation of R/G/B light-emitting monomers (Fig. 6a)^48^. The content of red, green and blue monomer materials can be changed to obtain different emitting lights, including white light. The EL spectrum and the commission internationale de l’Éclairage (CIE) coordinates of the devices based on these copolymers are shown in Fig. 6b, c. In addition to polymer molecules, small molecules that can emit white light have also been developed for SEL–WOLEDs, e.g., triphenylamine derivatives. Li, X. et al. reported a small molecule, tris(4-(phenylethynyl)phenyl) amine (TPEPA), with the characteristic adjustable photoluminescence (PL) spectrum covering almost the entire visible-light band, which was controlled by annealing temperature (Fig. 6d)^16^. When the annealing temperature is reached, the benzene ring and bis(phenylethylnyl)benzene in the organic molecular structure rotate, which changes the energy level and forms different species that emit blue, green, and red colours (Fig. 6e). By controlling the annealing temperature and applying the annealed thin films as the emitting layer, they created SEL–WOLEDs with a maximum EQE of 3.1% and a CIE coordinate of (0.3023, 0.3184) at a luminance of over 1000 cd m^−2^. The white light EL spectra of these devices and their CIE coordinates are shown in Fig. 6f, g, respectively.

### WQLEDs based on a single emissive layer

In the above cases, it is obvious that SEL–WOLEDs offer many unique advantages, such as simplified device structure, shorter fabrication process and lower cost. Undoubtedly, QD-based SEL–WQLEDs have also been valued and developed for these advantages. Moreover, compared with the multistep synthesis of organic emitters and high-precision doping in the vacuum thermal evaporation process, the low-cost synthesis and the colloidal solution feature of QDs give sufficient feasibility and advantages for SEL–WLEDs^62–66^. Therefore, some SEL–WQLEDs have emerged in recent years^21–25^.

**Fig. 7:**
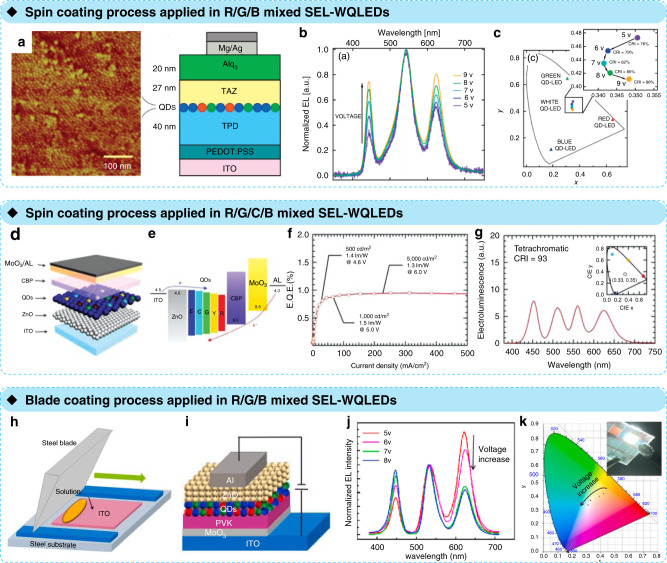
Mixing multi-colour QDs as a monolayer for SEL-WQLEDs. **a** Atomic force microscopy phase image of blue QDs forming approximately 1.1 monolayers on top of a 40-nm-thick TPD film and device cross section of a white QD–LED. **b** Normalised EL spectra of a white QD–LED for a set of increasing applied voltages. The relative intensities of the red and blue QD spectral components increase in comparison to the green QD component at higher biases. **c** CIE coordinates with the red, green, and blue QD–LEDs (triangles). The circle symbols show the evolution of the CIE coordinates and CRI of the white QD–LEDs upon increasing the applied bias. Reprinted with permission from ref. ^25^. Copyright 2007 American Chemical Society. **d** Device architecture and **e** energy-band diagram of white QLEDs with an inverted device structure of ITO/ZnO nanoparticle films (50 nm)/mixed QD-active layers/CBP (40 nm)/MoO_3_ (10 nm)/Al (100 nm). ZnO and QD layers were prepared by spin-casting, and CBP, MoO_3_, and Al were thermally evaporated on top of the spin-cast ZnO/QD layers. **f** EQE and **g** EL spectra of tetrachromatic (B + C + Y + R) white QLEDs. The brightness, power efficiency, and applied voltage of white QLEDs at brightness levels of 500, 1000, and 5000 cd m^−2^ are also indicated on EQE vs. J graphs. The CIE coordinate is displayed in the inset. Reprinted with permission from ref. ^22^. Copyright 2014 WILEY-VCH Verlag GmbH & Co. KGaA, Weinheim. **h** Schematic illustrating the experimental setup by blade coating. **i** Structure-mechanism diagram of WQLEDs. **j** Normalised EL spectra of white QLEDs at various voltages. **k** CIE coordinates of the device at various voltages; the inset is a photograph of the luminous white QLED at 8 V. Reprinted with permission from ref. ^24^. Copyright 2020 Elsevier

Anikeeva P. O. et al. formed a spin-coated SEL–WQLED by mixing R (CdSe/ZnS)/G (ZnSe/CdSe/ZnS)/B (ZnCdS) QDs as a monolayer (Fig. 7a)^25^. By simply optimising the R/G/B ratio (1:2:10) in the film, they created a device that exhibited a maximum EQE of 0.36% at 5.0 V and a CIE coordinate of (0.35, 0.41) at a luminance of approximately 100 cd m^−2^. The white EL spectrum and the CIE coordinates as a function of voltage are shown in Fig. 7b, c. Because of the adjustable spectrum and the colloidal solution characteristics of QDs, a series of multicolour QDs can be mixed together to form a single-layer film (Fig. 7d). The energy-level gradient of these QDs enables carrier injection and exciton recombination in these QLEDs (Fig. 7e); these include dichromatic QLEDs (blue/453 nm + yellow/562 nm), trichromatic QLEDs (blue/453 nm + green/545 nm + red/624 nm), and tetrachromatic QLEDs (blue/453 nm + cyan/513 nm + yellow/562 nm + red/624 nm)^22^. Especially in the tetrachromatic model, the SEL–WQLED emitted high bright natural white light with a CIE coordinate of (0.33, 0.35) and a high colour rendering index (CRI) of 93 (Fig. 7f, g).

QDs have potential for use in low-cost SEL–WLEDs due to both their synthesis process and device preparation. Not only the above spin-coating process but also the blade-coating process is valued because of its advantages in large-scale fabrication. A larger all-solution-processed SEL WQLED was demonstrated in a very recent work by Zeng Q. et al.^24^. By blade coating, the hole transport layer (PVK), the light-emitting layer (mixed R/G/B QDs), and the electron-transport layer (ZnO) were deposited sequentially (Fig. 7h, i). As shown in Fig. 7j, k, the device performed white light with a CIE coordinate of (0.33, 0.36) and a high CRI of 90 at a very high luminance (>10,000 cd m^−2^ @ 7 V). More noteworthy is that a 3 × 8-cm^2^ SEL–WQLED with homogenous white light emission was demonstrated by a blade-coating process, showing great potential in low-cost lighting and display.

### WPeLEDs with a single emissive layer

**Fig. 8:**
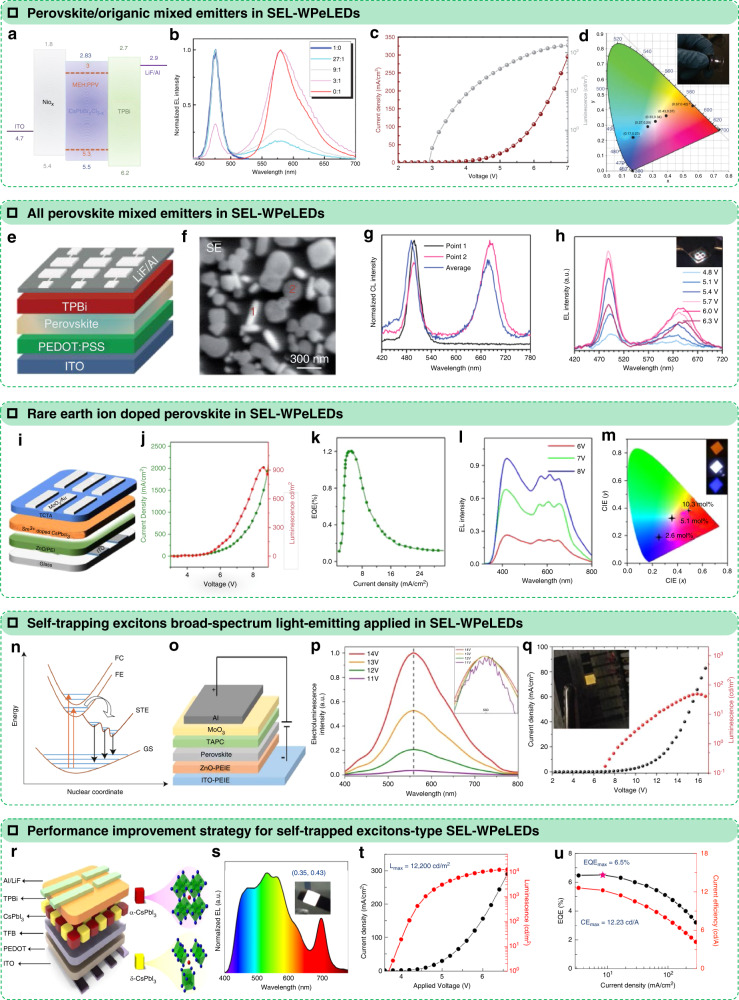
Perovskite emitters as multicolour centres in SEL-WPeLEDs. **a** Schematic band structure, **b** EL spectra, **c** J–L–V curve, and **d** CIE coordinate of CsPbBr_x_Cl_3−x_ nanocrystal and MEH:PPV-blend white LED. Reprinted with permission from ref. ^83^. Copyright 2017 WILEY-VCH Verlag GmbH & Co. KGaA, Weinheim. **e** Device architecture of the SEL–WPeLED. **f, g** Secondary-electron (SE) image and cathodoluminescence (CL) spectra of the BCPX film acquired at 5.0 keV. **h** EL spectra at different driving voltages (inset: photograph of a working device). Reprinted with permission from ref. ^87^. Copyright 2020 The Royal Society of Chemistry. **i** Schematic of the Sm^3+^ ion-doped CsPbCl_3_ PeLED configuration. **j** Current densities and luminescence, **k, l** EQE−J curve and EL spectra against voltage of PeLEDs based on 5.1 mol % Sm^3+^ ion-doped CsPbCl_3_ PeNCs. **m** CIE coordinates for the PeLED based on Sm^3+^ ion-doped CsPbCl_3_ PeNCs with different doping concentrations. Insets of **i**: photographs of PeLEDs with different Sm^3+^ ion-doping concentrations. Reprinted with permission from ref. ^27^. Copyright 2020 American Chemical Society. **n** A schematic illustration of STE emission. FC, free carrier state; FE, free exciton state; STE, self-trapped exciton state; GS, ground state. Reprinted with permission from ref. ^93^. Copyright 2020 Nature Publishing Group. **o** The electroluminescent device structure, glass/PEIE modified ITO/PEIE modified ZnO (20 nm)/Cs_2_Ag_0.60_Na_0.40_InCl_6_ film (50 nm)/TAPC (40 nm)/MoO_3_ (8 nm)/Al (100 nm), where PEIE is polyethylenimine, TAPC is 4,4′-cyclohexylidenebis [N,N-bis(4-methylphenyl) benzenamine]. **p** Electroluminescence spectra at applied voltages of 11 V, 12 V, 13 V, and 14 V. The inset is the normalised spectra. **q** Dependence of current density and luminance on the driving voltage. Reprinted with permission from ref. ^26^. Copyright 2018 Nature Publishing Group. **r** Structure of the perovskite WLED with an active layer composed of α-CsPbI_3_ and δ-CsPbI_3_. **s** Typical electroluminescence spectra of the perovskite WLEDs, which cover the whole visible band very well. **t** Current density–voltage (J–V) curve and luminance–voltage (L–V) curve of the Pe-WLEDs. **u** External quantum efficiency and current efficiency of the WLEDs. Reprinted with permission from ref. ^28^. Copyright 2021 Nature Publishing Group

Perovskite emitters have become desirable materials in recent years because of their excellent photoelectric properties, such as their high photoluminescence quantum yields (PLQYs) and bipolar carrier mobility, and they have great potential in lighting and displays^67–74^. Many PeLEDs have been demonstrated with EQEs higher than 20% in the green^75–77^, red^78,79^, and near-infrared^80–82^ regions. In addition to these exciting results, WPeLEDs, especially SEL–WPeLEDs, have also been developed rapidly^26–29,83–87^. In 2016, just after some of the earliest reports of PeLEDs, a SEL-WPeLED (Fig. 8a) was fabricated by blending a blue-perovskite emitter (CsPbBr_x_Cl_3−x_ nanocrystals) with orange-polymer materials (MEH: PPV)^83^. Blue–orange composite white light with CIE coordinates of (0.33, 0.34) can be obtained when the B/O weight ratio is 9:1 and the device is driven under 8 V, as shown in Fig. 8b–d. In addition, perovskite as an SEL with different components has also been proven to achieve white electroluminescence, and the device structure is shown in Fig. 8e^87^. In this device, an organic material (benzamidine hydrochloride, BHCl) successfully controlled the perovskite precursor solution to form segregated CsPb(Br_1−*x*_Cl_*x*_)_3_ and CsPb(Br_1−*y*_I_*y*_)_3_ grains (Fig. 8f), which act as a sky blue centre (498 nm) and a red centre (684 nm), respectively (Fig. 8g). This strategy resulted in an SEL–WPeLED with a balanced dual-colour white EL (Fig. 8h) and provided very useful guidelines for creating all-perovskite SEL–WPeLEDs. In another work from Sun R. et al., a rare-earth element (Sm) was introduced into the ABX_3_ perovskite structure, and a new single component—a samarium-doped perovskite material (CsPbCl_3_:Sm^3+^ nanocrystals)—was formed^27^. The colour of the PL or EL can be shifted from the blue to orange region by increasing the Sm^3+^ ion-doping concentration. When CsPbCl_3_:Sm^3+^ nanocrystals were employed as the single-emitting layer for fabricating SEL–WPeLEDs (Fig. 8i), the device showed a maximum luminance of 938 cd m^−2^ at 8.3 V and a maximum EQE of 1.2% (Fig. 8j, k). In the white light spectrum, this device maintained complete visible-light coverage and did not change under different voltages, showing potential in lighting with a CIE coordinate of (0.32, 0.31) and a CRI of 93 (Fig. 8l, m).

In addition to the monochromatic emitting characteristics of perovskites, as generally considered, their broadband-spectrum luminescence performance has also attracted the attention of researchers. As we proposed above, a single layer with broadband-light emission is considered to be an ideal strategy to achieve SEL–WLEDs, e.g., perovskite materials with the characteristics of self-trapped excitons (STEs)^26,28,29,84,86^. STEs are often generated in organic molecular crystals, condensed rare gases, and halide crystals owing to lattice distortion^88,89^. The strong carrier–phonon and exciton–phonon couplings of STE-type materials lead to lattice distortions that trap the carriers and excitons^90^. After high-energy excitation, electrons are promoted to excited states, rapidly fall into various self-trapped states, cause a large Stokes shift, and result in broadband-spectrum light emission^91,92^.

In 2018, Luo J. et al. developed a lead-free halide double perovskite and demonstrated broadband warm white light from this material (Cs_2_Ag_0.6_Na_0.4_InCl_6_)^26^. They used a metal-doping strategy to improve the luminescence characteristics of STEs in this perovskite emitter (Fig. 8n)^93^. Warm white light emission with a high PLQY of 86% was achieved owing to the efficient STE-type light emission. However, the SEL–WPeLED that used a Cs_2_Ag_0.6_Na_0.4_InCl_6_ film as the emitting layer only had a low current efficiency of 0.11 cd A^−1^ with a maximum luminance of less than 100 cd m^−2^ (Fig. 8o–q). In another work by Chen J. et al., a heterophase synergistic photoelectric effect was proposed to achieve high-efficiency electroluminescence from STEs^28,94–96^. By a controllable phase transition of perovskite CsPbI_3_ QDs, the *α*-phase and *δ*-phase were evenly distributed in the single-layer CsPbI_3_ film and exhibited a synergistic photoelectric effect. In this film, which comprised two coexisting phases, the *α*-CsPbI_3_ helped the *δ*-CsPbI_3_ achieve carrier transport and injection in the electric field due to its strong carrier-transport capacity. In contrast, *δ*-CsPbI_3_ is usually a poor carrier injector because it is confined due to STE. Using the *α*-/*δ*-CsPbI_3_ film that had two coexisting phases, Chen, J. et al. constructed a SEL–WPeLED (Fig. 8r) and achieved an effective broadband white EL spectrum with a CIE of (0.35, 0.43) at a high luminance of more than 1000 cd cm^−2^ (Fig. 8s). In this device, *α*-CsPbI_3_ can not only overcome the recombination obstacle of STE and achieve broadband white light emission but also complement the spectrum, which is missing part of the red region, by its intrinsic excitation. Benefitting from the photoelectric synergistic effect of *α*-/*δ*-CsPbI_3_, this SEL–WPeLED gave a maximum luminance of over 12000 cd cm^−2^, a maximum EQE of 6.5% at a low bias (4.6 V), and a maximum current efficiency of over 10 cd A^−1^ (Fig. 8t, u). Some perovskite-like materials such as caesium copper halides (CsCu_2_I_3_/Cs_3_Cu_2_I_5_) have also been developed in WPeLEDs owing to their STE-based broadband light emission from an SEL^29,84,86^. In a very recent work, Chen H. et al. introduced an organic additive into CsCu_2_I_3_/Cs_3_Cu_2_I_5_, resulting in trap-state reduction and PLQY promotion from 18% to 30%^29^. This strategy enhanced the EQE of caesium copper halide-based WPeLEDs to 3.1% with a high luminance of 1570 cd m^−2^ @ 5.4 V and warm white light emitting with CIE coordinates of (0.44, 0.53).

These SEL–WPeLEDs, especially based on the characteristics of STEs, show outstanding advantages in high-efficiency and broadband-spectrum white light emission. They will possess great potential in enabling low-cost commercialisation in lighting and other optoelectronics applications.

## Broad-spectrum emitters’ advances in SEL–WLEDs

In the above review of SEL–WLEDs, some broad-spectrum emitters, mainly those made from a single material, have attracted much attention because they can achieve multicolour or broadband-spectrum coelectroluminescence throughout the entire visible light region. It is obvious that these broad-spectrum emitters have distinct advantages for low-cost and simple processes and are the most commercially acceptable for lighting, displays, and many other applications. Broad-spectrum emitters are able to overcome the obstacles faced by current technology: (i) complicated manufacturing processes, including epitaxial growth for GaN-based WLEDs and thermal vacuum evaporation with precise doping for WOLEDs; (ii) material costs of current WLEDs that are high due to the synthesis process (e.g., organic emitters); (iii) differences in emitters involving material stability and emitting stability, e.g., balanced regulation of exciton recombination between different colours, different physical–chemical properties, and uncoordinated emitting characteristics between different emitters. Therefore, broad-spectrum emitters formed through solution processes, such as perovskite emitters, provide a feasible path to WLEDs while promising lower costs, better compatibility, and multiscene adaptability.

Here, we summarise the performance of some SEL–WLEDs that include broad-spectrum emitters, which are generally materials that possess the coelectroluminescence of multicolour centres (e.g., triphenylamine derivatives, CsPbCl_3_: Sm^3+^, *δ*-CsPbI_3_, and CsCu_2_I_3_), as shown in Table 1. In the last two decades, following the development of OLEDs, some copolymers and small molecules have been developed for SEL–WOLEDs^16,38,48,49,61^. Single-emitting layers formed by spin coating (SC) or vacuum thermal evaporation (VTE) have been proven to achieve multicolour coemission and high brightness. More notably, perovskite emitter-based SEL-WLEDs have appeared only in the last two or three years, but they have shown outstanding performance in broadband white emission and have a high luminous efficiency^27–29,97^. Perovskite emitters can be obtained from a simple synthesis process and abundant raw materials, as well as a full solution process that includes both material preparation and emitting-layer preparation, making them bright prospects in the future development of SEL–WLEDs.

**Table 1:** Summary of the performance of SEL–WLEDs containing broad-spectrum emitters

Cases	Broad-spectrum emitters	Film process	EL range (nm)	CIE/CRI	Max. luminance (cd m^−2^)	Max. EQE (%)	Year
Organic emitters	3,5-dimethyl-2,6-bis(dimesitylboryl)-dithie-no[3,2-b:2′,3′-d]thiophene	SC	400–750 B/G/R	(0.31, 0.42)/–	3800 @ 18 V	0.35	2005^49^
	Poly-fluorene copolymer (PG3R2)	SC	400–800 B/O	(0.34, 0.37)/–	820 @ 11 V	–	2005^48^
	4,4′-di(9-(10-pyrenylanthracene))triphenylamine (DPAA)	VTE	450–700 B/O	(0.29, 0.34)/–	12320 @ 8 V	–	2008^38^
	R/G/B fluorene copolymers (PF-Cz-G-R)	SC	400–800 B/G/O	(0.29, 0.30)/–	3512 @ 12 V	3.39	2013 ^61^
	tris(4-(phenylethynyl)phenyl)amine (TPEPA)	VTE	400–750 B/O	(0.30,0.32)/72.3	2200 @ 13 V	3.12	2019^16^
Perovskite emitters	2D (PEA)_2_PbCl_2_Br_2_	SC	400–700 Broadband	(0.22,0.32)/–	70 @ 7 V	–	2018^97^
	CsPbCl_3_: Sm^3+^ nanocrystals	SC	400–800 Broadband	(0.32,0.31)/ 93	938 @ 8.7 V	1.2	2020^27^
	*α*/*δ*-CsPbI_3_ QDs	SC	400–750 Broadband	(0.35,0.43)/90	12200 @ 6 V	6.5	2020^28^
	CsCu_2_I_3_/Cs_3_Cu_2_I_5_ mixed film	SC	390–740 Broadband	(0.44, 0.53)/–	1570 @ 5.4 V	3.1	2021^29^

## Outlook

### Light-extraction strategies for SEL–WLEDs

As we listed in Table 1, some SEL–WLEDs with broad-spectrum emitters that exhibit high performance in spectrum and brightness have been developed. However, their luminescence performance is relatively lower than that of traditional WLEDs. A feasible strategy is applying light-extraction methods to reduce the inherent light loss of their planar-device structure, which has been confirmed to trap nearly 80% of light in its structure owing to the different optical properties (refractive index) of its materials, these properties include the substrate mode, waveguide mode, and plasmon mode^98–100^. Therefore, some light-extraction strategies to increase light output have been proposed and have achieved a great improvement in the luminescence performance of OLEDs^101–105^, QLEDs^106–108^, and PeLEDs^109–111^. Huang Y. et al. applied a nanocomposite substrate to reduce the light loss in waveguide and substrate modes, achieving an EQE of more than 38% in a WOLED-device structure^103^. Wang S. et al. realised a variety of patterns by nanoimprint methods in a green QLED, and the EQE can be increased from 11.13% to 13.45% compared with the planar EQE; this increase demonstrates their importance in improving luminescence performance, as well as the importance of many other nanostructures^107^. Some very recent works confirmed that light extraction is also applicable to perovskite LEDs, and a synergetic outcoupling enhancement strategy in PeLEDs was reported by Shen Y. et al. with the EQE improved from 13.4% to 28.4%^110^. Because this method of light extraction has an outstanding ability to improve the luminescence performance of various OLEDs, QLEDs, and PeLEDs, it will also play an important role in improving the luminescence efficiency of SEL–WLEDs. Although relevant results are rarely reported, it is worth studying and exploring the application of light extraction in SEL–WLEDs.

### Optoelectronic properties of the emitters in SEL–WLEDs

The performance of these SEL–WLEDs (Table 1) is limited not only by the structure of the devices but also by the optoelectronic properties of the emitters, which include the light-emitting mechanism, structure regulation, and synthesis strategy. We believe that future studies will focus on two key factors: the internal quantum efficiency (IQE) and the operational stability of these emitters and SEL–WLEDs. For example, although the efficiency of monochromatic devices that use perovskite emitters is relatively high, the efficiency of WLEDs is far from sufficient. Some strategies for improving the IQE need to be further developed, such as crystal-structure regulation, defect passivation, and interface engineering between the SEL and transport layers. Debjit M. et al. utilised an element-doping strategy to regulate the crystal structure of lead-free double-perovskite NCs (CsAgIn_1−x_Bi_x_Cl_6_) for use as perovskite emitters^112^. The PLQE can achieve a twofold increase through optimisation of the doping concentration of Bi. In SEL–WPeLEDs, Chen H. et al. introduced an organic additive (Tween) into the precursor solutions to reduce the trap states, which can facilitate the growth of high-quality crystals and then lead to a PLQE of 30%, which is higher than 18% PLQE of the control sample^29^. In terms of photoelectric characteristics, a photoelectric synergistic effect in an α-/δ-CsPbI_3_ single-layer heterophase film was also revealed in Chen J. et al.’s work, suggesting a representative strategy for improving the carrier-injection ability in STE-based WPeLEDs^28^. Greater effort to improve the carrier injection capability will become an important trend for highly efficient SEL–WPeLEDs. Meanwhile, the stability of perovskites and the lifetime of PeLEDs have always been key limitations. WLEDs with lifetimes of thousands of hours are needed for commercial applications. However, lifetime research on SEL–WLEDs is still rare, and long-lifetime SEL–WLEDs have not yet been verified. Therefore, the device performance and lifetime will be an important direction in the future development of SEL–WLEDs. Fortunately, these issues are attracting the attention of researchers. In a study by Luo J. et al., the breakthrough of STEs in Cs_2_Ag_0.6_Na_0.4_InCl_6_ greatly improved the PLQY to 86% with outstanding stability (over 1000 h)^26^. Some stability studies of monochromatic light PeLEDs will also inspire stability research on SEL–WPeLEDs. Some combined and feasible strategies have been developed for PeLEDs, and perovskite emitters are limited by their instability caused by hydrooxygen, thermal, electric fields, etc. The essential reason is believed to come from the perovskite itself and its ion-crystal characteristics, such as easy ion migration, trends of electrochemical reaction and interfacial reaction^113^. Some very recent processes are working on reducing defects^75^, improving thermal stability^114^, enhancing spectral stability under an electric field^79^, and preventing various chemical reactions of perovskites^115^. Some long-lifetime optoelectronic devices made with perovskite materials have been realised in recent studies, e.g., a long half-lifetime of 682 hours in formamidinium-based PeLEDs^115^ and over 1000 h (lifetime: T_90_) in perovskite-based solar cells^116,117^. These works have inspired stability research on PeLEDs and will also promote the development of long-lifetime perovskite-based WLEDs.

### Developing broad-spectrum emitters for SEL–WLEDs

In the past few decades, many cases of SEL–WLEDs have been confirmed and have demonstrated performance comparable to conventional multilayer WLEDs. However, most of these high-performance SEL–WLEDs use mixed multicolour organic emitters, which face the same problems as traditional WOLEDs, such as the need for vacuum evaporation equipment, high-precision doping, and material cost. Therefore, some broad-spectrum emitters, especially those that can be manufactured by the solution method, have advantages in overcoming these problems and will become ideal emitters for SEL–WLEDs. For example, some very recent studies on perovskites have shown their potential in this area, but the multicolour/broadband light-emitting properties of these perovskite emitters come from different luminescence mechanisms, such as energy transfer in some ion-doped perovskites and self-trapping excitons in some heterogeneous perovskite emitters. Therefore, future exploration of their luminescence mechanism will be an important research direction.

### Spectral design and regulation in SEL–WLEDs

The different spectral characteristics of SEL–WLEDs determine their application field. In lighting applications, SEL–WLEDs with a broadband white light spectrum are an ideal choice because they can cover the entire visible-light region and are able to imitate sunlight very well. In displays, SEL–WLEDs that possess multiple narrow peaks or controllable discolouration of red, green, and blue colours will play an important role. For example, Sun R. et al. achieved the coemission of multiple colours by introducing Sm^3+^ into CsPbCl_3_ nanocrystals^27^. Benefitting from the multilevel-excitation characteristics of Sm^3+^, the CsPbCl_3_:Sm^3+^-based SEL–WPeLEDs emitted white light, including intrinsic blue light (410 nm) from CsPbCl_3_ and yellow–red light (565 nm, 602 nm, and 645 nm) from Sm^3+^. Their work showed that broad-spectrum emitters with adjustable spectral characteristics, combined with a low-cost solution process, will make SEL–WLEDs an ideal choice for lighting, displays, and other applications. In addition to the energy transfer mechanism and multicolour coemission of these ion-doped perovskites, continuous efforts are needed to introduce various rare-earth ions into the perovskite structure to achieve more adjustable multicolour luminescence. SEL–WLEDs with R/G/B standard colour and pure white light quality will be valuable in both lighting and displays. For instance, combining SEL–WLED with a colour filter will be a feasible strategy for creating microdisplays while reducing the cost and energy consumption.
